# Single-site ^123^I-FP-CIT reference values from individuals with non-degenerative parkinsonism—comparison with values from healthy volunteers

**DOI:** 10.1186/s41824-020-0074-2

**Published:** 2020-03-13

**Authors:** Rachid Fahmi, Günther Platsch, Alexandre Bani Sadr, Sylvain Gouttard, Stephane Thobois, Sven Zuehlsdorff, Christian Scheiber

**Affiliations:** 1 Siemens Medical Solutions USA, Inc., Molecular Imaging, Knoxville, TN USA; 2 Siemens Healthineers, Erlangen, Germany; 3 Nuclear Medicine, Hospices Civils de Lyon, 69500 Bron, France; 4 Movement Disorder Clinic, Pierre Wertheimer Neurologic Hospital, Hospices Civils de Lyon, 69500 Bron, France; 5 Faculté de Médecine Lyon Sud, Université Claude Bernard Lyon 1, Lyon, France; 6 Institut des Sciences Cognitives Marc Jeannerod, UMR 5229, CNRS, Bron, France

**Keywords:** Dopamine transporter, Reference values, ^123^I-FP-CIT SPECT,·Age effect, Gender difference

## Abstract

**Purpose:**

Iodine 123-radiolabeled 2β-carbomethoxy-3β-(4-iodophenyl)-*N*-(3-fluoropropyl) nortropane (^123^I-FP-CIT) SPECT can be performed to distinguish degenerative forms of movement disorders/parkinsonism/tremor from other entities such as idiopathic tremor or drug-induced parkinsonism. For equivocal cases, semi-quantification and comparison to reference values are a necessary addition to visual interpretation of ^123^I-FP-CIT scans. To overcome the challenges of multi-center recruitment and scanning of healthy volunteers, we generated ^123^I-FP-CIT reference values from individuals with various neurological conditions but without dopaminergic degeneration, scanned at a single center on the same SPECT-CT system following the same protocol, and compared them to references from a multi-center database built using healthy volunteers’ data.

**Methods:**

From a cohort of 1884 patients, we identified 237 subjects (120 men, 117 women, age range 16–88 years) through a two-stage selection process. Every patient had a final clinical diagnosis after a mean follow-up of 4.8 ± 1.3 years. Images were reconstructed using (1) Flash3D with scatter and CT-based attenuation corrections (AC) and (2) filtered back projection with Chang AC. Volume-of-interest analysis was performed using a commercial software to calculate specific binding ratios (SBRs), caudate-to-putamen ratios, and asymmetry values on different striatal regions. Generated reference values were assessed according to age and gender and compared with those from the ENC-DAT study, and their robustness was tested against a cohort of patients with different diagnoses.

**Results:**

Age had a significant negative linear effect on all SBRs. Overall, the reduction rate per decade in SBR was between 3.80 and 5.70%. Women had greater SBRs than men, but this gender difference was only statistically significant for the Flash3D database. Linear regression was used to correct for age-dependency of SBRs and to allow comparisons to age-matched reference values and “normality” limits. Generated regression parameters and their 95% confidence intervals (CIs) were comparable to corresponding European Normal Control Database of DaTscan (ENC-DAT) results. For example, 95% CI mean slope for the striatum in women is − 0.015 ([− 0.019, − 0.011]) for the Flash3D database versus − 0.015 ([− 0.021, − 0.009]) for ENC-DAT. Caudate-to-putamen ratios and asymmetries were not influenced by age or gender.

**Conclusion:**

The generated ^123^I-FP-CIT references values have similar age-related distribution, with no increase in variance due to comorbidities when compared to values from a multi-center study with healthy volunteers. This makes it possible for sites to build their ^123^I-FP-CIT references from scans acquired during routine clinical practice.

## Introduction

Single photon emission computed tomography (SPECT) imaging with iodine 123-radiolabeled 2β-carbomethoxy-3β-(4-iodophenyl)-*N*-(3-fluoropropyl) nortropane (^123^I-FP-CIT or ^123^I-ioflupane, brand name: DaTscan™, GE Healthcare) is a widely used nuclear imaging method to assess the integrity of the presynaptic dopaminergic system by measuring dopamine active transporters’ (DATs) availability in the striatum (Booij et al. 1997a; Neumeyer et al. 1994). Knowing whether a patient, exhibiting movement disorders or parkinsonian signs, presents with degeneration of the dopaminergic system has major implications in terms of diagnosis, prognosis, and care (Thobois et al. 2019; O’sullivan et al. 2008). However, the clinical diagnosis of a parkinsonian syndrome remains sometimes challenging and inconclusive, especially at disease onset and for patients with atypical clinical presentation or those taking drugs that might induce per se the concomitant neurological abnormalities (Thobois et al. 2019; Catafau and Tolosa 2004). A normal ^123^I-FP-CIT SPECT excludes the diagnosis of Parkinson’s disease as mentioned in the revised diagnosis criteria for Parkinson’s disease (Postuma et al. 2015). In addition, ^123^I-FP-CIT is valuable in distinguishing idiopathic Parkinson’s disease (PD) and Parkinson “plus” syndromes (reduced radiotracer binding) from essential tremor (ET), psychogenic, post-neuroleptic or vascular parkinsonism, and dopa-responsive dystonia (normal radiotracer binding) (Booij et al. 1997b; Booij et al. 2001; Booth et al. 2015). ^123^I-FP-CIT has also been proven as valuable in differential diagnosis of dementia with Lewy body (DLB) and Alzheimer’s disease (AD) (Thobois et al. 2019; Booth et al. 2015; Walker et al. 2002).

In clinical settings, ^123^I-FP-CIT images are routinely assessed by expert readers based on visual evaluation using a 0 to 3 grading system according to the visual scale proposed by Catafau et al. (Catafau and Tolosa 2004). The reproducibility of this technique has been questioned (Tondeur et al. 2010), in particular for cases that are equivocal or exhibit an early onset of the disease. Furthermore, visual interpretation can be especially challenging in a follow-up scenario to differentiate relevant changes from irrelevant ones. In such difficult-to-interpret cases, region-based quantification is often advised to be used in combination with visual interpretation (Darcourt et al. 2010; Djang et al. 2012) as it can help clinicians reach a more confident interpretation of the scan and increases confidence in less experienced readers (Söderlund et al. 2013; Albert et al. 2016). Semi-quantification was also shown to add value to visual analysis of ^123^I-FP-CIT in differential diagnosis of DLB from AD (Nicastro et al. 2017). These semi-quantification methods use either manually drawn or predefined regions of interest. However, quantitative measures have little clinical value without reference ranges, especially in cases when diagnostic decisions cannot be drawn from quantification as the scans cannot be interpreted visually as abnormal (Albert et al. 2016). In addition, as ^123^I-FP-CIT uptake depends on normal ageing, quantitative values are only of value if age effect on DAT availability is accounted for (Nicastro et al. 2016).

A few years ago, a ^123^I-FP-CIT database was generated from healthy volunteers in a multi-center European study (Varrone et al. 2013). This study included 139 healthy individuals (74 men, 65 women; age range 20–83 years, mean 53 ± 19 years) scanned at 13 different centers on 17 different SPECT systems of 11 different models. To address inter-center variability, quantitative values for each SPECT system were corrected using camera-specific calibration factors generated through phantom experiments (Koch et al. 2006; Tossici-Bolt et al. 2011; Dickson et al. 2012). Despite the efforts to minimize inter-site variability, there remained significant variability in specific binding ratio (SBR) which could not be explained by age and other considered covariates and which might be related to SPECT equipment, including hardware and reconstruction software, used in the European Normal Control Database of DaTscan (ENC-DAT) study as was noted in (Buchert et al. 2016a). This led to some inconsistencies when comparing data and may have resulted in higher variability in the estimation of the regression lines, modelling the uptake decline as a function of age, and that of the percentage declines per decade as was noted by the authors. Two semi-quantitative methods were used to calculate reference values from both corrected and non-corrected data, and corresponding outcomes were compared to each other. Depending on the quantification method, the study showed a significant decline of DAT availability between 4 and 6.7% per decade. Regression analyses were performed, and outcomes were presented according to gender, showing a significant gender effect on uptake ratios in caudate and putamen when using non-corrected data and when using only attenuation corrected data. Significant effects of both age and gender on striatal SBRs were observed for all data, corrected and uncorrected, when a semi-quantification method accounting for partial volume effect was used.

In order to cope with the challenge of recruiting healthy volunteers and multi-center variability, Nicastro et al. (Nicastro et al. 2016) proposed an approach to calculate site-specific reference values using scans from individuals with non-degenerative conditions scanned following the same protocol. A normal scan was defined as a grade 0 ^123^I-FP-CIT SPECT (Catafau and Tolosa 2004). This study included a cohort of 182 subjects with an older age range compared to the ENC-DAT database (73 men and 109 women (60%); age range 40–93 years, mean 69.1 ± 11.2 years). Most of the included subjects had drug-induced parkinsonism (DIP) (44%), while the remaining subjects had essential tremor (ET) (21%), psychogenic parkinsonism (17%), or other non-parkinsonian conditions. Not all included subjects were followed over time, and final neuropathological assessment was unavailable for the majority of them. Therefore, it may be possible that this study included cases with pre-clinical degenerative conditions affecting the nigrostriatal system.

Reference limits were generated using an automated semi-quantitative software, and age-dependent reference limits were established based on the percentile approach. Corresponding outcomes were compared to a manual analysis method. The study has also shown a linear effect of age on striatal uptake decrease of 6.8% per decade. This effect was accounted for using linear regression which helped to determine age-dependent reference limits of SBRs. The authors have also shown greater decline of uptake in women than men in all striatal regions, yet none of these differences in slope was statistically significant. Hence, identical reference values were established for both genders. In comparison with the results from the ENC-DAT study (Varrone et al. 2013), Nicastro et al. (Nicastro et al. 2016) found slightly higher intercepts and greater slopes, but comparable mean caudate and putamen SBR values according to gender.

In order to address some of the limitations mentioned above, we propose to build a database of reference values for ^123^I-FP-CIT using a large cohort of subjects across a wide age range and with a well-balanced gender representation. Each included subject had both a normal ^123^I-FP-CIT SPECT scan, based on semi-quantification and on visual interpretation by two trained nuclear medicine physicians, as well as a clinical diagnosis of non-degenerative parkinsonism or other non-parkinsonian entities at baseline and confirmed after a follow-up period ranging from 3.5 to 10 years by experienced neurologists. Imaging data were all acquired at the same center on the same SPECT-CT imaging system and following the same imaging protocol. We have established reference values and limits for the striatum, caudate nucleus, putamen, anterior putamen, and posterior putamen while accounting for the age effect on DAT availability. We computed regression parameters and their 95% confidence intervals and compared them to parameters from the ENC-DAT study. C/P ratios and asymmetry values were also calculated as additional parameters that could be useful to assess the integrity of the nigrostriatal pathway. The robustness of the generated reference values and limits was tested using a cohort of 22 patients with mixed diagnoses.

## Materials and methods

**Fig. 1:**
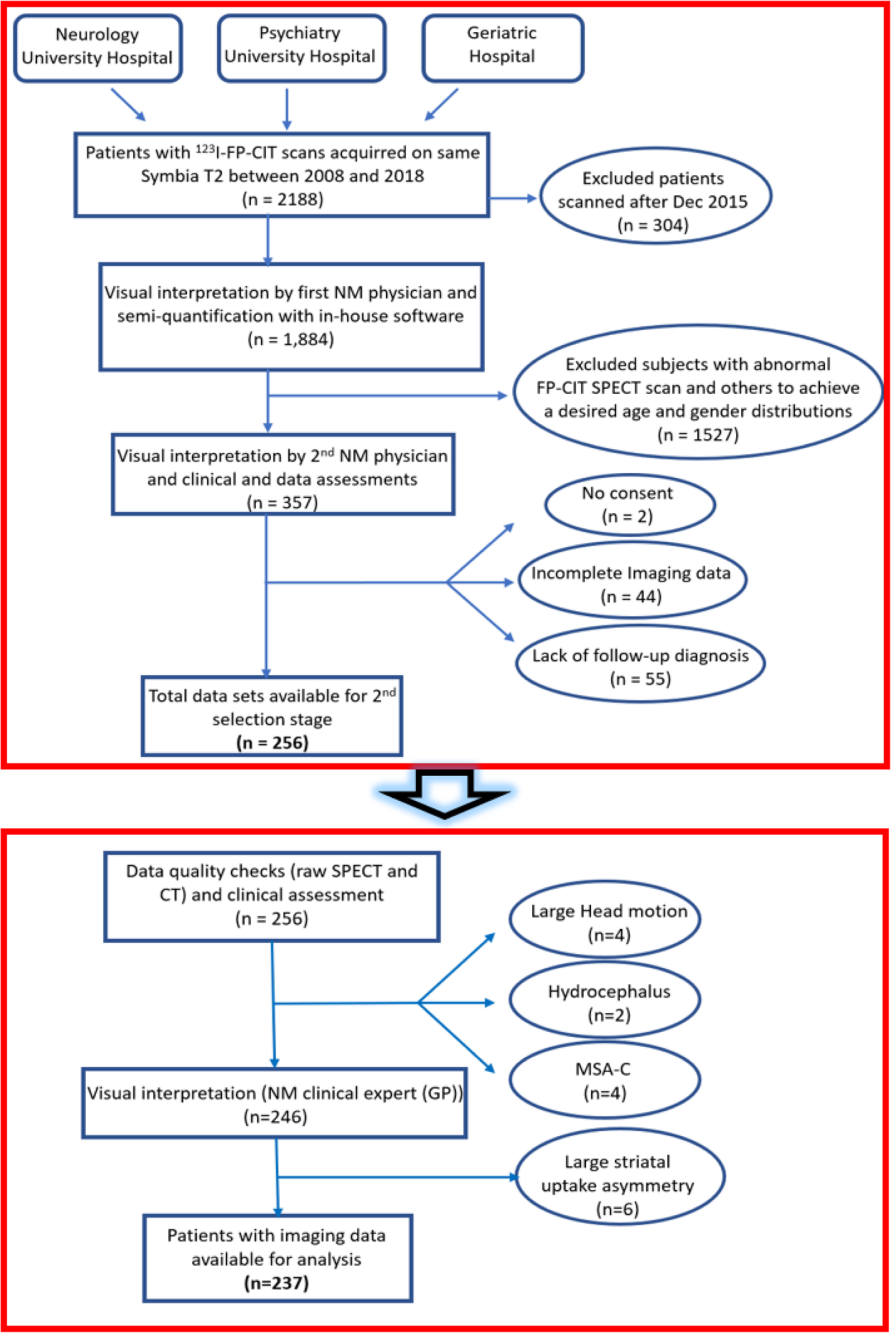
Diagram showing the two-stage selection procedure for patients’ selection and inclusion in the present study. The top panel corresponds to stage 1 (clinical site, Lyon, France), and the bottom panel corresponds to the second selection stage (Siemens Medical Solutions USA, Inc., Molecular Imaging, Knoxville, TN). MSA-C cerebellar multi-system atrophy

The selection of our cohort was performed in two different stages as illustrated on the diagram shown in Fig. 1. In the first stage, selection was based on visual reads combined with semi-quantification with an in-house software, and assessment of clinical diagnoses. This stage was performed at Lyon University Neurological Hospital nuclear medicine department where data were collected (CS and ABS, Lyon, France). The second stage consisted of additional and rigorous quality control of both anatomical and functional imaging data, combined with visual reads and semi-quantification of the SPECT data with *syngo*.via® software. Patients’ clinical information was also taken into consideration when making a decision to include or reject a patient at this second selection stage. This stage was performed by RF and GP. Following are detailed descriptions of these selection stages.

### Patient selection—stage 1

Every patient included in the present study was evaluated by a neurologist from Lyon (France) university hospital’s departments of neurology, psychiatry, or geriatrics and then referred to the nuclear medicine imaging center for a ^123^I-FP-CIT examination. The aim of this ^123^I-FP-CIT SPECT was to determine whether the patients’ abnormal movements (tremor mostly) or parkinsonian syndrome were related to a degeneration of the nigrostriatal dopaminergic pathway or not. More precisely, these patients exhibited atypical tremor, parkinsonian syndrome, or dementia.

A pool of 1884 ^123^I-FP-CIT scans, performed on the same Symbia T2 scanner (Siemens Medical Solutions USA, Inc.) between January 2008 and December 2015, was available. We consecutively assessed all available scans, one at a time by the order of their scan date. Scan “normality” was mainly determined by visual interpretation and confirmed by semi-quantification using a fully automated in-house analysis program (CS). This program was implemented based on routines from the statistical parametric mapping (SPM) toolbox^1^, with CT-based attenuation correction (CTAC) and correction for age effect on DAT availability. Note that this analysis program is independent of the software application implemented in *syngo*.via®, which was used to generate the reference values. Once a scan was classified as normal, a second NM physician (ABS) was asked to visually confirm its normality. Then, we assessed the integrity of corresponding imaging and clinical data and the availability of final diagnosis based on a follow-up of at least 3 years after the ^123^I-FP-CIT SPECT scan was acquired. As the number of normal SPECT scans increased, new inclusions were further segregated to achieve a targeted normal age distribution and a balanced gender distribution. Subjects selected at this stage were contacted to get their written informed consent to use their anonymized data including for commercial purposes.

### Patient selection—stage 2

The anonymized clinical and imaging data of the 256 subjects selected in stage 1 were further quality-checked (GP) looking for any large patient motion in the SPECT projections and/or any reconstruction or imaging artifacts, as well as any anatomical abnormalities (e.g., major hydrocephalus or atrophies) in the CT images. Additional visual and semi-quantitative assessments of the SPECT data were performed (RF and GP) using a striatal analysis methodology implemented in *syngo*.via® software looking for any uptake abnormalities, such as large asymmetries. This software application generates quantifiable measures as well as a parametric “slab” view of the patient’s image for improved visual assessment (Buchert et al. 2016b). The final clinical diagnoses as well as the clinical reads, performed at stage 1, of all SPECT images were considered when selecting which subjects to include in the database.

### SPECT imaging: data acquisition and reconstruction

Patients were scanned on the same Symbia™ T2 system equipped with low-energy high-resolution collimators. Scans started 3 h post-intravenous injection of ~ 185 MBq of ^123^I-FP-CIT which occurred 1 h following thyroid blockade with perchlorate when needed. In every case, the patient’s head was constrained with a head holder to minimize motion, and acquisition was performed with the following parameters: rotational radius kept between 13 and 15 cm; matrix 128 × 128; 120 projection angles over 360°; and a hardware zoom of 1.23 × 1.23 to achieve an in-plane pixel size of 3.9 × 3.9 mm^2^. In addition to the photopeak imaging window (159 keV ± 8%, 147–171 keV), two additional scatter energy windows were acquired. For each patient, a diagnostic CT was acquired (with a CT dose index of 35 mGy, and a dose length product of 940 mGy*cm) and was used for attenuation correction (AC). We generated two databases of reference values corresponding to two image reconstructions methods: (1) Flash3D (OSEM3D with resolution recovery) with 10 iterations and 8 subsets, CTAC (as recommended for OSEM reconstructions), a triple energy window method for scatter correction, and 8-mm FWHM Gaussian post-reconstruction smoothing; and (2) filtered back projection (FBP) reconstruction with Chang attenuation correction (linear correction coefficient: *μ* = 0.11 cm^− 1^), and Butterworth filter of order 5 and a cut-off frequency of 0.45 cycles/pixel.

### Image quantification and analyses

**Fig. 2:**
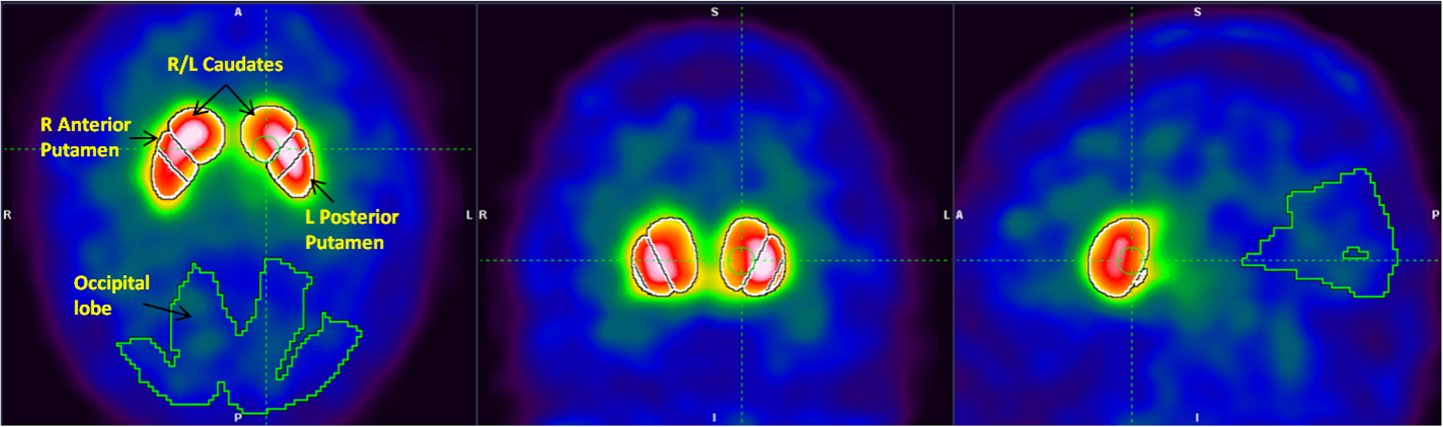
Axial, coronal, and sagittal views showing predefined striatal and background volumes of interest overlaid on a spatially and intensity normalized ^123^I-FP-CIT image. R right, L left

We used *syngo.*via® software to calculate^2^ reference values on twelve striatal volumes-of-interest (VOIs), including the left and the right sides of the caudate, striatum, putamen, anterior putamen, and posterior putamen. For each VOI, left and right specific binding ratios (SBRs) and asymmetry values as well as C/P ratios^2^ were generated as follows: first, each ^123^I-FP-CIT image was automatically registered to a SPECT template in the Montreal Neurological Institute (MNI) space using an affine transformation, followed by (automatic) positioning of predefined striatal and background (occipital lobe) VOIs. To ensure accurate positioning of these VOIs on the spatially normalized SPECT images, manual adjustments (translations and rotations) of the automatically placed VOIs, in three planes, were performed whenever needed. Figure 2 shows an example of the predefined striatal and background VOIs overlaid on a spatially and intensity normalized ^123^I-FP-CIT image. The automatic SPECT-based registration to MNI space is sensitive to striatal uptake and to background noise characteristics and hence could be suboptimal for some challenging cases. In a few such cases, we used a CT-based registration method to guide the spatial normalization process of the SPECT image.

For each striatal VOI, left and right SBR values were calculated as:

1 $$ \mathrm{SBR}=\left({C}_{\mathrm{voi}}-{C}_{\mathrm{occip}}\right)/{C}_{\mathrm{occip}} $$

where *C*_voi_ and *C*_occip_ are the mean uptakes of the 75% hottest voxels within a side of the striatal VOI and within the occipital lobe, respectively. The asymmetry values, between corresponding left and right VOIs, were calculated using the following formula:

2 $$ \mathrm{asym}\ \left(\%\right)=200\times \mathrm{abs}\ \left({\mathrm{SBR}}_{\mathrm{L}}-{\mathrm{SBR}}_{\mathrm{R}}\right)/\left({\mathrm{SBR}}_{\mathrm{L}}+{\mathrm{SBR}}_{\mathrm{R}}\right), $$

where SBR_R_ and SBR_L_ denote the SBR values computed on the left and the right sides of a given striatal VOI, respectively. We have also calculated the left and the right C/P ratios for the striatum, caudate, and putamen.

### Comparison of new subjects to reconstructed databases

In order to test the robustness of the generated reference values, we have compared a set of subjects with different diagnoses against the generated databases. A total of 22 subjects (9 normal and 13 with confirmed degenerative forms of parkinsonism) were used for this purpose. For a test subject of a given age and for each striatal VOI, the patient’s left and right SBRs were separately compared to the corresponding predicted and age-matched reference SBR. The latter is calculated by substituting the subject’s age in the corresponding regression line equation. Using the equation of the lower bounding line of the predictive interval (− 95% PI), the patient’s age-matched normality threshold is also calculated and used to classify the subject. Left and right C/P ratios as well as asymmetries were also compared against corresponding references. A patient was classified as normal if each of its measured parameters falls within the corresponding normal range defined by the normality thresholds.

### Statistical analyses

For each striatal VOI, differences between corresponding left and right SBR values were evaluated with a paired *t* test. A multivariate analysis was used to investigate the effects of age and gender on SBR values, with the dependent variable being the SBR. The relationship between age and SBR for both genders was analyzed using linear regression. Regression parameters are reported with their 95% confidence intervals (95% CI). For each regional SBR, the regression line, defined as *y*_r_ = slope × age + intercept, served to calculate age-matched references, and the standard error (SE) of the fitting model was used to derive the model’s ± 95% prediction interval (PI) as *y* = *y*_r_ ± 2 × SE. The PI represents the range where SBR values of a subject with normal striatal uptake (calculated in a similar way as the generated reference values) has a probability of 95% to fall within. The lower bounds of these intervals (i.e., − 95% PI given by *y*_l_*= y*_r_ – 2 × SE) are used as reference limits for SBR values. That is, a subject whose scan is acquired and analyzed following the same protocols is suspected of having dopaminergic dysfunction if one of the SBR values is below the corresponding − 95% PI line (i.e., a value that is more than 2 × SE below corresponding age-matched reference).

Normality of measured outcomes was assessed using Normal Q–Q plots which revealed a normal distribution of all SBR distributions, whereas the asymmetries and the C/P ratios were normalized using the box-cox power transformation method prior to assessing the effect of age and gender on them.

Finally, the difference between the *r* values of two regression fits, *r*_1_ and *r*_2_, was evaluated using the following formula (Cohen et al. 2014):

3 $$ z=\frac{\left({z}_1-{z}_2\right)}{\sqrt{\frac{1}{\left({n}_1-3\right)}+\frac{1}{\left({n}_2-3\right)}}} $$

where *z*_1_ and *z*_2_ refer to the Fisher’s *r* to *z* transformation of the coefficients *r*_1_ and *r*_2_ and *n*_1_ and *n*_2_ denote the corresponding sample sizes. Significance was set at *p* = 0.05.

Statistical analyses were mainly performed using the Statistics Toolbox of Matlab R2014a (MathWorks, Natick, MA).

## Results

### Patient selection

Stage 1 of the patients’ selection process initially resulted in identifying 357 eligible patients who were subsequently contacted to get their written informed consent to be included in the study.

Further checks resulted in 101 exclusions: 44 subjects were excluded due to incomplete imaging data files. Additional 55 subjects were excluded due to lack of their follow-up diagnoses, and two subjects did not consent to be included in the study. Finally, we retained 256 subjects: 20 of whom were young (age range 23–57 years) with attention deficit hyperactivity disorder (ADHD) diagnosis (see the “Discussion” section), and the remaining 236 patients had a final diagnosis of non-degenerative parkinsonism, other movement disorders, or epilepsy based on long-term follow-ups ranging from 3 to 10 years (mean 4.8 ± 1.3 years) (Rizzo et al. 2016).

The second selection stage resulted in the exclusion of 19 additional subjects for the following reasons: scans of four subjects had large head motion, two patients had major hydrocephalus which rendered the positioning of the predefined striatal VOIs on their SPECT images very challenging, three patients were imaged using different collimators, four patients without “evidence of dopaminergic deficit or SWEDD” had a final diagnosis of MSA-C and hence excluded by our clinical expert (GP), and six patients had large ^123^I-FP-CIT uptake asymmetries and were then considered as abnormal scans. In total, 237 subjects (120 men and 117 women, age 62.2 ± 15.7 years, range 16–88 years) were included in the present study. Most included subjects had either ET or DIP (49.8%). The young patient sub-group (8.5%) with ADHD was included to have references at young ages. Table 1 summarizes final clinical diagnoses for all included subjects.

**Table 1:** Final diagnoses of subjects included in the generated databases

Diagnostic	Ratio (%)	*n*
Essential tremor	27.0	64
Drug-induced parkinsonism	22.8	54
Dementia without dopaminergic degeneration (AD or frontotemporal dementia)	10.5	25
Miscellaneous or mixed (rheumatologic disorders, myopathy, primary lateral sclerosis, epilepsy)	10.1	24
Attention deficit hyperactivity disorder	8.5	20
Psychiatric disorders, psychogenic parkinsonism, or movement disorders	6.3	15
Vascular parkinsonism without dopaminergic degeneration	6.1	14
Normal pressure hydrocephalus	2.5	6
Dystonia	1.3	3
Restless legs syndrome	1.3	3
Amyotrophic lateral sclerosis	0.8	2
Epilepsy	0.8	2
Orthostatic tremor	0.8	2
Genetic parkinsonism	0.8	2
Cerebellar ataxia	0.5	1
Total	100	237

Differences in specific binding ratio between hemispheres

No statistical differences were found between left and right SBR values for any striatal VOI irrespective of gender, nor between left and right SBRs from men and women considered separately. Hence, for each striatal VOI, the left and the right SBRs were averaged to estimate a single reference value. Left and right SBR values for the Flash3D database are presented in Table 2. Corresponding results for the FBP database are not shown. Averaged left and right SBRs are shown in Table 4 for both databases and genders.

**Table 2:** Comparison between SBRs in both hemispheres for the Flash3D database according to gender

	Striatum	Caudate	Putamen	Anterior putamen	Posterior putamen
All					
Right	3.05 ± 0.49	3.12 ± 0.49	2.96 ± 0.51	3.11 ± 0.53	2.75 ± 0.52
Left	3.01 ± 0.48	3.09 ± 0.49	2.91 ± 0.50	3.04 ± 0.52	2.74 ± 0.53
*p* value	0.36	0.46	0.27	0.14	0.64
Men					
Right	2.97 ± 0.42	3.03 ± 0.43	2.88 ± 0.44	3.03 ± 0.47	2.67 ± 0.44
Left	2.95 ± 0.44	3.02 ± 0.44	2.85 ± 0.47	2.99 ± 0.47	2.66 ± 0.51
*p* value	0.76	0.87	0.62	0.5	0.87
Women					
Right	3.14 ± 0.54	3.21 ± 0.54	3.04 ± 0.56	3.19 ± 0.57	2.83 ± 0.58
Left	3.07 ± 0.52	3.15 ± 0.52	2.97 ± 0.54	3.09 ± 0.55	2.79 ± 0.55
*p* value	0.35	0.41	0.3	0.17	0.63

Values are mean ± SD. The *p* value indicates the statistical significance between left and right SBRs (paired *t* test). *SBRs* specific binding ratios

A one-way ANOVA test comparing the averaged right and left SBR values for different diagnostic subgroups (see Table 1) resulted in no significant differences for caudate, putamen, and striatum (*p* > 0.05). These results are shown in Table 3 for the FBP database. Similar results were found for the Flash3D database but are not shown here.

**Table 3:** Averaged left and right SBR values corresponding to different diagnostic groups

Diagnostic group	Caudate	Putamen	Striatum
Essential tremor (*n* = 64, age 64.7 ± 13.1, range 19–87)	2.64 ± 0.40 (1.63, 3.64)	2.47 ± 0.39 (1.60, 3.40)	2.57 ± 0.39 (1.62, 3.54)
Drug-induced parkinsonism (*n* = 54, age 62.4 ± 13.4, range 25–86)	2.58 ± 0.44 (1.82, 3.80)	2.41 ± 0.42 (1.73, 3.62)	2.51 ± 0.43 (1.83, 3.72)
Dementia without dopaminergic degeneration (*n* = 25, age 71.8 ± 9.5, range 55–87)	2.57 ± 0.34 (1.94, 3.52)	2.42 ± 0.35 (1.63, 3.33)	2.5 ± 0.34 (1.81, 3.44)
Miscellaneous or mixed (*n* = 24, age 62.0 ± 13.1, range 36–82)	2.55 ± 0.31 (2.11, 3.34)	2.40 ± 0.34 (1.86, 3.33)	2.49 ± 0.32 (2.02, 3.33)
Attention deficit hyperactivity disorder (*n* = 20, age 37.2 ± 9.8, range 22–56)	3.06 ± 0.33 (2.55, 3.64)	2.91 ± 0.30 (2.39, 3.53)	3.00 ± 0.31 (2.48, 3.52)
Psychiatric disorders, psychogenic parkinsonism, or movement disorders (*n* = 15, age 59.7 ± 18.6, range 25–84)	2.82 ± 0.55 (2.09, 4.06)	2.69 ± 0.53 (1.76, 3.76)	2.76 ± 0.54 (1.96, 3.93)
Vascular parkinsonism without dopaminergic degeneration (*n* = 14, age 72.7 ± 11.4, range 46–88)	2.39 ± 0.45 (1.73, 3.08)	2.19 ± 0.47 (1.48, 2.81)	2.30 ± 0.45 (1.62, 2.91)
Others (*n* = 21, age 62.7 ± 17.2, range 16–85)	2.60 ± 0.35 (1.87, 3.24)	2.41 ± 0.33 (1.73, 2.99)	2.52 ± 0.33 (1.81, 3.11)
*p* value^a^	0.244	0.078	0.144

^a^*p* values are adjusted for age. Values are mean ± SD. SBR values in parentheses define the SBR range for each VOI

### Gender and age effects on specific binding ratio

**Fig. 3:**
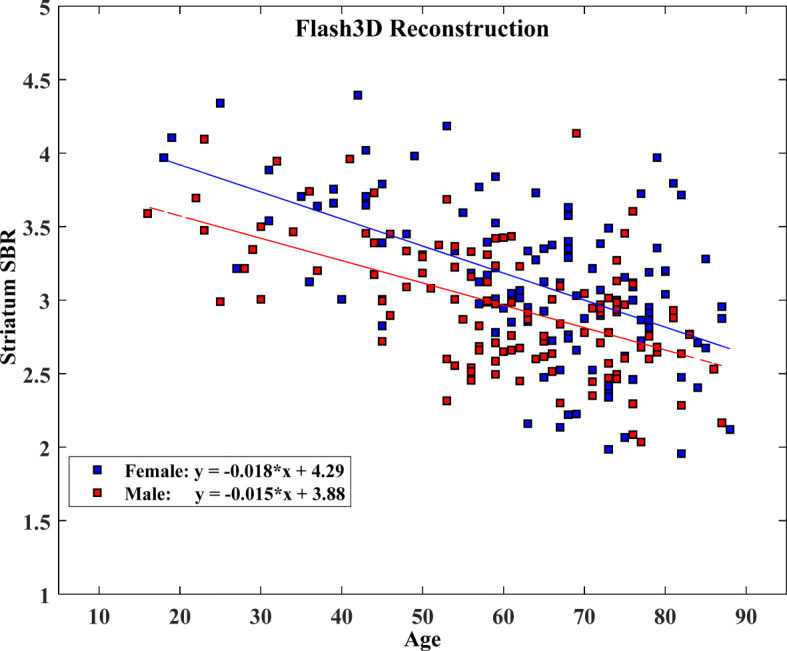
Effect of age and gender on specific binding ratio (SBR) values computed as the average of left and right SBRs within the striatum and plotted as a function of age. Also shown are regression lines with slope and intercept for the Flash3D database (female, blue; male, red)

**Fig. 4:**
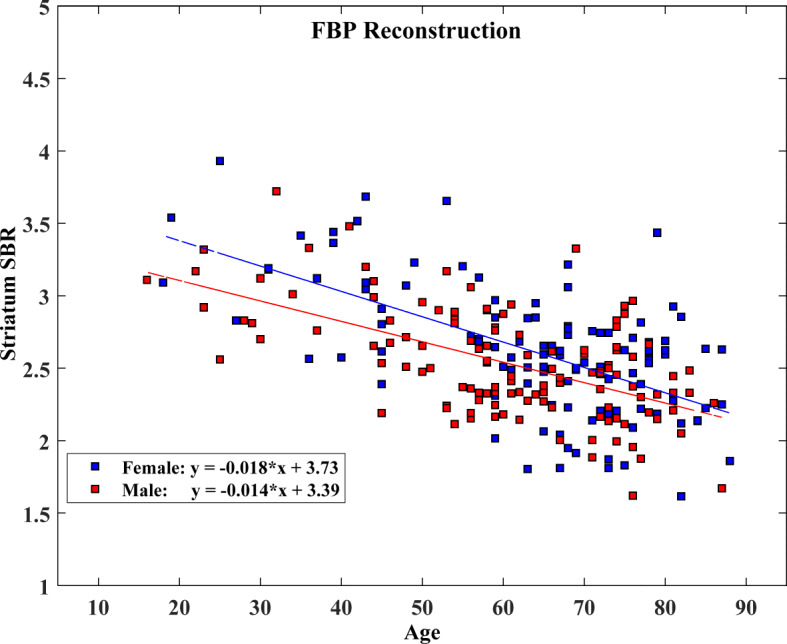
Effect of age and gender on specific binding ratio (SBR) values computed as the average of left and right SBRs within the striatum and plotted as a function of age. Also shown are regression lines with slope and intercept for the FBP database (female, blue; male, red)

We found that women had slightly higher SBRs with greater variances compared to men in all striatal regions regardless of the reconstruction method. Figures 3 and 4 show SBRs measured within the striatum according to gender for the Flash3D and the FBP databases, respectively, as a function of age. This difference tends to decrease with ageing. The differences in SBR between genders were only statistically significant for the Flash3D database as shown in Table 4. Hence, we have established SBR reference values identical for men and women for both reconstructions.

**Table 4:** Average of left and right SBRs for Flash3D and FBP databases, for men, women, and for combined genders

	Flash3D			FBP		
	Striatum	Caudate	Putamen	Striatum	Caudate	Putamen
Men (*n* = 120)	2.96 ± 0.42	3.03 ± 0.43	2.87 ± 0.44	2.53 ± 0.38	2.60 ± 0.39	2.43 ± 0.38
Women (*n* = 117)	3.11 ± 0.52	3.18 ± 0.52	3.00 ± 0.54	2.61 ± 0.45	2.68 ± 0.45	2.52 ± 0.45
All (*n* = 237)	3.03 ± 0.48	3.10 ± 0.48	2.93 ± 0.50	2.57 ± 0.41	2.64 ± 0.42	2.48 ± 0.42
*F* value						
Age	55.19 (*p* < 0.001)	34.66 (*p* < 0.001)	53.85 (*p* < 0.001)	65.57 (*p* < 0.001)	60.88 (*p* < 0.001)	67.02 (*p* < 0.001)
Gender	5.63 (*p* = 0.018)	6.89 (*p* = 0.009)	5.35 (*p* = 0.022)	2.20 (*p* = 0.14)	1.99 (*p* = 0.16)	2.45 (*p* = 0.12)

Values are mean ± SD. *F* values and corresponding *p* values are presented for the effects of age and gender on SBR. *SBRs* specific binding ratios

Conversely, the effect of age was statistically significant for both databases and all SBRs as shown on Table 4. We observed a consistent decrease of SBR values with age for men and women in all striatal VOIs (e.g., Figs. 3 and 4). Parameters of the linear regression analysis of SBR as a function of age and their 95% CIs for both databases are presented in Table 5 for all striatal VOIs, irrespective of gender. Linear regression analysis revealed that measured SBRs in the striatum, for both genders combined, decreased by 30.54% (Flash3D) and 33.39% (FBP) within the considered age range (16–88 years). This corresponds to a reduction rate in striatum SBR of 4.24% (Flash3D) and 4.64% (FBP) per decade. Women showed a steeper decline with advancing age compared to men in all striatal regions. For both men and women, posterior putamen showed the highest SBR decline while caudate SBR was the slowest to decline. The mean reduction rate of SBR was between 3.80 and 5.67% per decade for men and between 4.47 and 5.70% for women, depending on the used reconstruction method and on the striatal VOI. The calculated percentage declines of SBRs per decade are summarized in Table 6.

**Table 5:** Linear regression analysis of DAT availability versus age, irrespective of gender

Database	Regression parameters	Caudate	Putamen	Anterior putamen	Posterior putamen	Striatum
Flash3D	Intercept	4.004 (3.78, 4.23)	4.055 (3.84, 4.27)	4.134 (3.91, 4.36)	3.942 (3.73, 4.16)	4.028 (3.81, 4.24)
	Slope	− 0.0145 (− 0.018, − 0.011)	− 0.018 (− 0.021, − 0.015)	− 0.0171 (− 0.021, − 0.013)	− 0.0194 (− 0.023, − 0.016)	− 0.0166 6 (− 0.019, − 0.013)
	2 × SE	0.83	0.80	0.86	0.80	0.80
	*r*^2^	0.22	0.32	0.27	0.36	0.27
FBP	Intercept	3.585 (3.40, 3.77)	3.434 (3.26, 3.61)	3.541 (3.35, 3.73)	3.289 (3.12, 3.46)	3.521 (3.34, 3.7)
	Slope	− 0.0151 (− 0.018, − 0.013)	− 0.0154 (− 0.018, − 0.013)	− 0.0151 (− 0.018, − 0.012)	− 0.0159 (− 0.019, − 0.013)	− 0.0152 (− 0.018, − 0.012)
	2 × SE	0.68	0.67	0.71	0.64	0.66
	*r*^2^	0.32	0.34	0.30	0.37	0.33

Values in parentheses are 95 % confidence intervals for the intercepts and slopes. *FBP* filtered back projection, *DAT* dopamine active transporter, *SE* standard error of regression model

**Table 6:** Age-related decline in DAT availability (in % per decade)

	Flash3D		FBP	
	Men	Women	Men	Women
Striatum	4.24	4.74	4.59	5.23
Caudate	3.80	4.39	4.47	5.09
Putamen	4.94	5.23	4.85	5.33
Anterior putamen	4.47	4.92	4.53	5.12
Posterior putamen	5.64	5.67	5.33	5.7

*FBP* filtered back projection

**Fig. 5:**
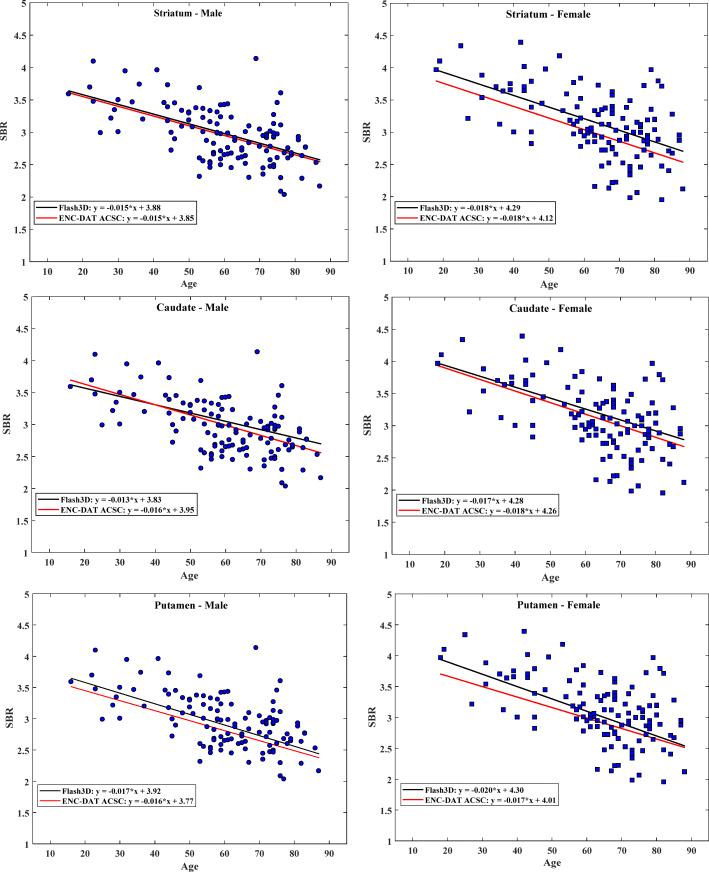
Comparison between SBR values from Flash3D database with values from the ENC-DAT study using BRASS analysis of uncalibrated attenuation and scatter corrected scans (ENC-DAT-ACSC), according to gender

We have compared regression parameters from the reconstructed Flash3D database to parameters generated from healthy volunteers by the ENC-DAT study using the attenuation and scatter corrected (ACSC) and uncalibrated scans processed with BRASS analysis method (Varrone et al. 2013). Corresponding results are shown in Table 7 and graphically displayed in Fig. 5. Using Eq. 3, we found no differences in *r* values between men and women for both databases. Similarly, no differences in *r* values were found between Flash3D database and the “uncalibrated ENC-DAT-ACSC” database according to gender in all striatal VOIs.

**Table 7:** Comparison of linear regression parameters from the generated Flash3D database with those from the ENC-DAT study using BRASS analysis of uncalibrated and ACSC data

	Men		Women	
	Flash3D	Uncalibrated ENC-DAT-ACSC	Flash3D	Uncalibrated ENC-DAT-ACSC
Striatum				
Intercept	3.88 (3.62, 4.13)	3.85 (3.51, 4.19)	4.29 (3.95, 4.63)	4.12 (3.74, 4.50)
Slope	− 0.015 (− 0.019, − 0.011)	− 0.015 (− 0.021, − 0.009)	− 0.018 (− 0.024, − 0.013)	− 0.018 (− 0.025, − 0.011)
*r*^2^	0.32	0.28	0.30	0.31
Caudate				
Intercept	3.83 (3.57, 4.11)	3.9 (3.58, 4.33)	4.28 (3.93, 4.63)	4.26 (3.86, 4.66)
Slope	− 0.013 (− 0.018, − 0.009)	− 0.016 (− 0.022, − 0.009)	− 0.017 (− 0.022, − 0.012)	− 0.018 (− 0.025, − 0.010)
r^2^	0.24	0.28	0.26	0.29
Putamen				
Intercept	3.92 (3.67, 4.17)	3.77 (3.43, 4.11)	4.30 (3.95, 4.64)	4.01 (3.62, 4.40)
Slope	− 0.017 (− 0.022, − 0.013)	− 0.016 (− 0.022, − 0.009)	− 0.02 (− 0.025, − 0.015)	− 0.017 (− 0.024, − 0.010)
*r*^2^	0.38	0.28	0.33	0.29

*ACSC* attenuation correction and scatter correction, *ENC-DAT* European Normal Control Database of DaTscan

### Asymmetries and caudate-to-putamen ratios

Linear regression analysis performed on the box-cox transformed asymmetries and C/P ratios revealed an age effect only on the putamen asymmetry for the Flash3D database (*y =* 0.003 × *x* + 1.19, *r*^2^*=* 0.018, SE = 0.37, *p* = 0.037). When comparing these parameters between men and women, the differences were not statistically significant.

The reference limit corresponding to each one of these parameters is estimated as the sample mean plus twice the sample standard deviation (i.e., mean and SD of all corresponding reference values in the database). For instance, the reference limit corresponding to striatum asymmetry is 3.02 + 2 × 2.41 = 7.84% (Flash3D) and 2.44 + 2 × 1.91 = 6.26 (FBP).

**Fig. 6:**
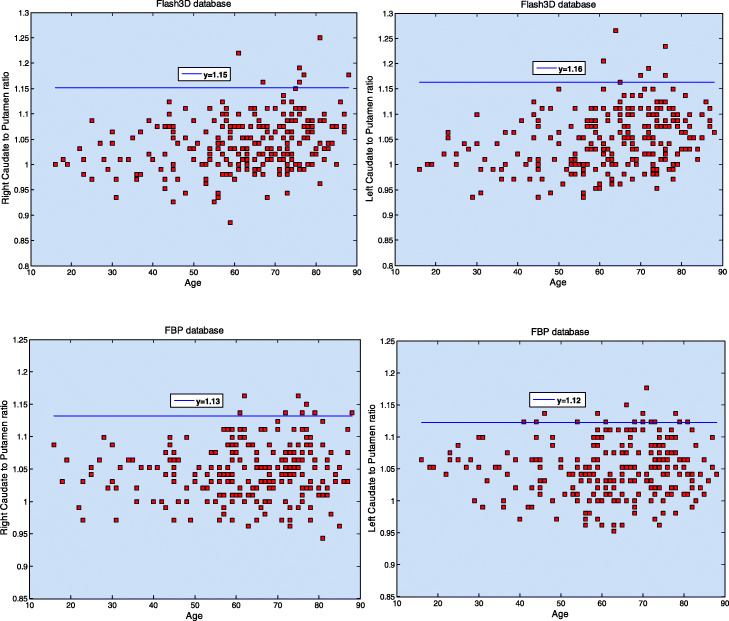
Left and right putamen to caudate ratios for the Flash3D (upper row) and the FBP (bottom row) databases irrespective of gender. The solid blue line (*y* = mean + 2 × standard deviations of corresponding reference values) defines the reference limit above which a scan could be suspected as being positive

**Fig. 7:**
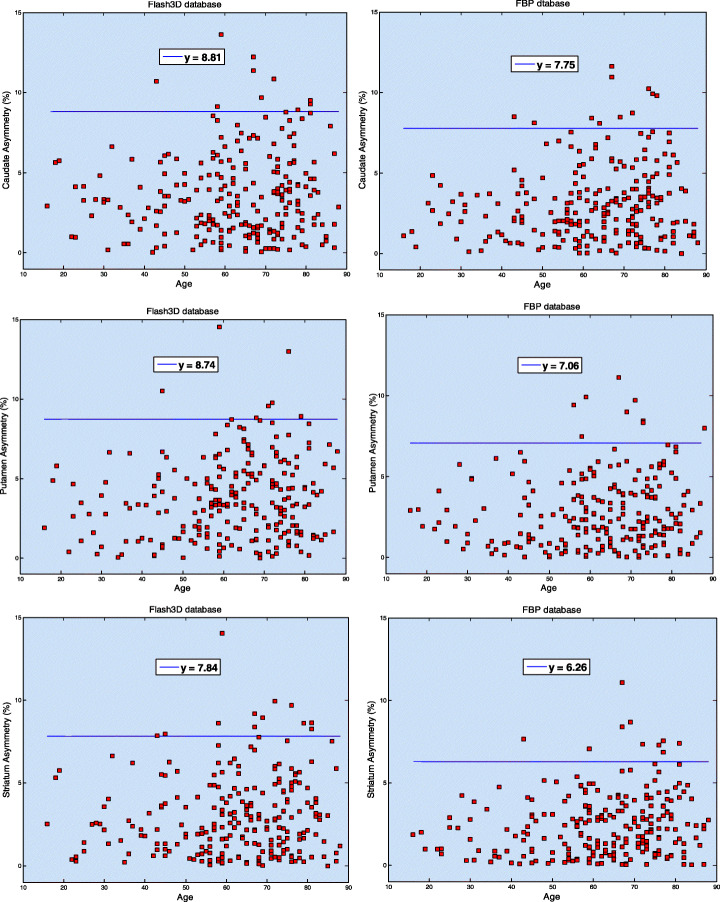
Asymmetry values (%) corresponding to caudate, putamen, and striatum (from top to bottom) and for the Flash3D (left) and FBP (right) databases. The solid blue line (*y* = mean + 2 × standard deviations of corresponding reference values) defines the reference limit above which a scan could be suspected as being positive

Sample means and SDs of the left and right C/P ratios and asymmetry values are summarized in Table 8 for both databases. Comparisons between C/P ratios corresponding to Flash3D database with those from the ENC-DAT study are presented in Table 9 for men and women. Figures 6 and 7 show scatter plots of left and right C/P ratios and asymmetry values, respectively, with horizontal lines defining reference limits.

**Table 8:** Left and right C/P ratios and asymmetry values for the Flash3D and the FBP databases

	Left C/P ratio	Right C/P ratio	Striatum asymmetry	Caudate asymmetry	Putamen asymmetry
Flash 3D	1.04 ± 0.05	1.04 ± 0.05	3.02 ± 2.41	3.56 ± 2.62	3.68 ± 2.53
FBP	1.05 ± 0.04	1.04 ± 0.04	2.44 ± 1.91	3.09 ± 2.33	2.73 ± 2.17

Asymmetries are given in %. Values are mean ± SD*. FBP* filtered back projection, *C/P* caudate-to-putamen ratio

**Table 9:** Comparison of C/P ratios from the Flash3D database with those from the ENC-DAT study with BRASS analysis of uncalibrated and ACSC data

	Men		Women	
	Left	Right	Left	Right
Flash3D	1.05 ± 0.05	1.05 ± 0.06	1.05 ± 0.05	1.04 ± 0.05
Uncalibrated ENC-DAT-ACSC	1.06 ± 0.12	1.09 ± 0.13	1.07 ± 0.11	1.09 ± 0.10

Values are given as mean ± SD. *C/P* caudate-to-putamen ratio, *ENC-DAT* European Normal Control Database of DaTscan, *ACSC* attenuation and scatter corrected images

### Comparison of new subjects to reconstructed databases

**Fig. 8:**
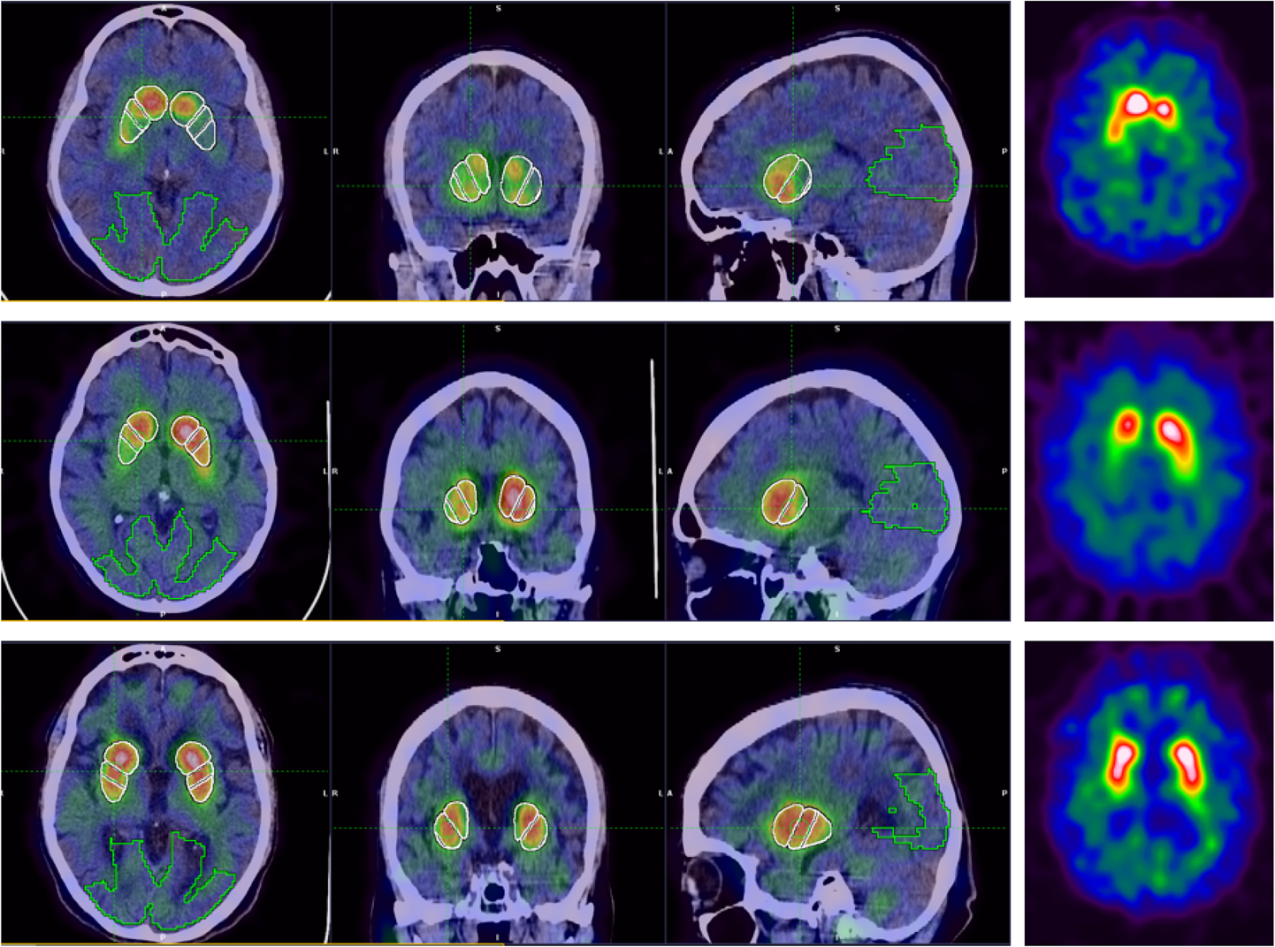
Examples of three test cases used to validate the generated databases: two positive (first and second rows) and one negative (third row) case. Each row from left to right, axial, sagittal, and coronal fused view of the the patient’s SPECT and CT images showing the striatal and occipital VOIs, and the corresponding slab view images (Buchert et al. 2016b) generated within the *syngo*.via® software and used for visual assessments

**Fig. 9:**
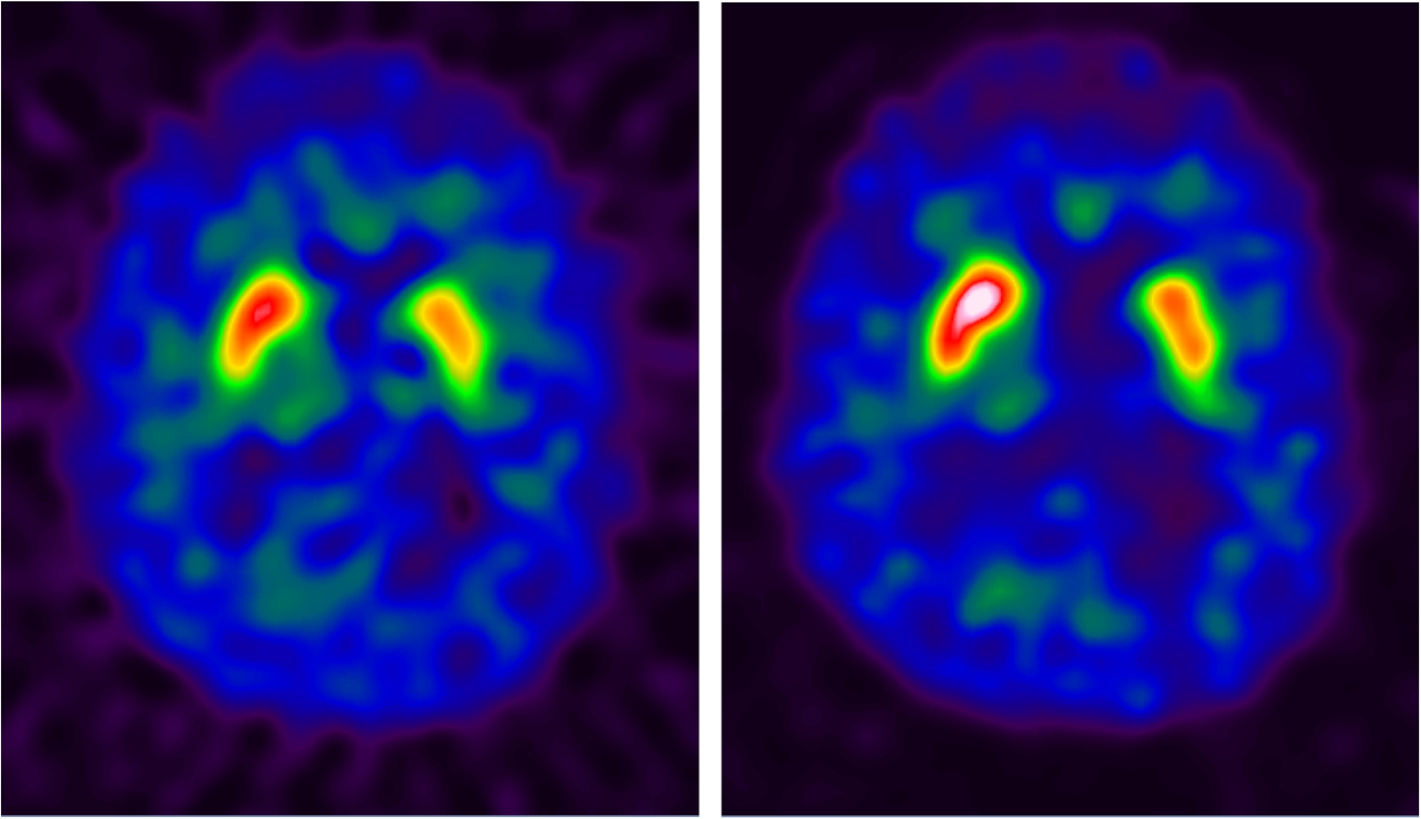
Slab view (Buchert et al. 2016b) corresponding to a 63-year-old male patient showing uniformly asymmetric uptake with a reduction on the entire left striatum. Left (FBP) and right (Flash3D) reconstructions

Scans belonging to the 22 test subjects were compared to the generated databases using the *syngo*.via® software. For all abnormal cases (13/13), and independent of the reconstruction method, one or more SBR values fell below corresponding age-matched reference limit confirming abnormality of the scans. For most of these cases, the C/P ratios and/or the asymmetry values were also greater than their corresponding reference limits. The majority of tested cases, including the nine normal ones, could easily be accurately classified based only on visual assessments of the corresponding parametric slab views (see examples on Fig. 8) (Buchert et al. 2016b). However, for a normal case corresponding to a 63-year old male subject, the tracer uptake was uniformly reduced on the left side compared to the right side as can be clearly seen on the slab view on Fig. 9 for both reconstructions. Without quantification, this may lead to visually interpreting this scan as abnormal or to reducing confidence in its visual interpretation. However, for this case, all calculated binding ratios and asymmetries were within normal ranges, with the lowest SBR being − 1.32 (Flash3D) and − 1.86 (FBP) SDs from age-matched mean. In addition, all asymmetries were within 2 × SDs from respective reference values for both reconstructions, with the caudate exhibiting the highest asymmetry for the Flash3D reconstruction (4.41% or 0.24 × SDs from sample mean). This shows the added value of quantifying and comparing scans to reference values.

## Discussion

In this study, we generated a large database of ^123^I-FP-CIT reference values from a cohort of patients with normal ^123^I-FP-CIT scans and no confirmed forms of degenerative parkinsonism at baseline nor after a follow-up ranging from 3 to 10 years. Our cohort was rigorously selected, in two separate stages, from a pool of patients’ data collected over 8 years at the same center and acquired on the same SPECT-CT imaging system following the same acquisition and processing protocols. This helped overcome the inter-center variability and the challenge of recruiting a large enough cohort of healthy volunteers as in the previously published European (*n* = 139) (Varrone et al. 2013) and Japanese (*n* = 256) (Matsuda et al. 2018) databases.

To the best of our knowledge, this is the largest cohort of patients with normal ^123^I-FP-CIT SPECT, and a well-balanced gender distribution (50.65% men and 49.35% women) across a wide age range (16–88 years). To better fit the age effect on tracer uptake at younger ages, we have included 20 young subjects (age range 23–57 years, mean 37 years) with ADHD, two siblings (17 and 30 years old) who were initially suspected of genetic parkinsonism but was not confirmed, and two young adults (25 and 31 years old) with psychogenic parkinsonism. The ADHD patients underwent a ^123^I-FP-CIT SPECT for research purposes given the controversy in the literature whether the dopaminergic system of patients with this disease is affected or not (van Dyck et al. 2002; Volkow et al. 2007; Costa et al. 2013). Discussions related to ADHD pathophysiology is outside the scope of this paper.

One originality of this study is that our “normal” data sets, as identified at the first stage of the patients’ selection process, went through additional and independent quality and medical checks followed by analysis using a dedicated commercial software that allows quantification and improved visual assessment of ^123^I-FP-CIT SPECT scans (Buchert et al. 2016b). At this second selection stage, we excluded 19/256 patients, four of which had normal ^123^I-FP-CIT scans and were diagnosed with MSA-C. These SWEDD cases represent a negligible proportion of our cohort (1.6%), in agreement with the findings by Nicastro et al. in their prospective study (Nicastro et al. 2018) where they showed that 3/155 (2.1%) subjects with suspected degenerative parkinsonism (1 corticobasal syndrome, 1 MSA-C, and 1 PD) and 1/53 (1.9%) with DLB had a normal visual ^123^I-FP-CIT SPECT.

Using a dedicated *Striatal Analysis* software (*syngo*.via®), we generated two different versions of the database corresponding to two reconstruction methods often used in clinical routine, and we investigated the effects of gender and age on all measured outcomes. Our results showed a significant linear effect of age on SBR values in all striatal volumes of interest, as was previously reported by others (e.g., (Nicastro et al. 2016; Varrone et al. 2013)). We obtained an overall rate of SBR decline per decade, averaged over all striatal VOIs and over both genders, of 4.80% (Flash3D) and 5.02% (FBP). This agrees with what has been reported in the literature for healthy volunteers (between 3.6 and 7.5% per decade) (Nicastro et al. 2016; Varrone et al. 2013; Matsuda et al. 2018; Pirker et al. 2000; van Dyck et al. 1995; van Dyck et al. 2002). In particular, the ENC-DAT study (Varrone et al. 2013) reported an average age-related decline in DAT availability of 5.5% per decade for both genders in good agreement with our measured decline. These results clearly validate our approach of using normal ^123^I-FP-CIT SPECT uptake values obtained in non-healthy subjects but without dopaminergic degeneration to generate a normal and age-stratified ^123^I-FP-CIT database. Several factors, including image reconstruction and correction as well as quantitative analysis, may have contributed to the variability in the estimated decline rate of DAT availability seen between different published studies. We also found that regardless of gender and irrespective of the reconstruction method, the rate of SBR decline was faster for putamen compared to that for caudate, and even more so in the posterior region of the putamen compared to that in the anterior region. There are conflicting results on this topic in the literature (Varrone et al. 2013; Matsuda et al. 2018; Eusebio et al. 2012; Kaasinen et al. 2015; Volkow et al. 2007; Nobili et al. 2013) which we believe deserves further investigations.

To describe the age-related effect on DAT availability, we used a linear model to best fit our data, as was performed in other studies (Nicastro et al. 2016; Varrone et al. 2013; Matsuda et al. 2018). A previous study using ^123^I-IPT SPECT (Mozley et al. 1996) on a small sample of healthy volunteers (*N* = 18, age range 19–67 years) has suggested that age-related decline is rapid during young adulthood, followed by slower decline throughout middle age, and reported that non-linear function provided better fit for age effect on DAT availability. In a different study using a larger cohort (*N* = 126) of healthy subjects and ^123^I-β-CIT SPECT (van Dyck et al. 2002), the authors suggested that linear models provide appropriate fit of DAT loss with ageing and used such a model to report an average decline per decade of 6.5%. The authors have also noted that second order polynomial was slightly better but nearly linear in the considered age range (18–88 years) which is similar to the age range of our cohort. In the present study, we have tested different fitting models (linear, quadratic, logarithmic, exponential, and power) and calculated correlation coefficients to evaluate their quality of fit. We found that, for most outcomes, the linear and the quadratic models are both equally superior compared to the other models. Hence, we decided to use the linear model to investigate the effect of age on our population and to compare our results against studies that used similar models.

Linear regression analysis was used to establish age-matched reference SBRs and their corresponding normality limits in the striatum, caudate, putamen, anterior putamen, and posterior putamen. This is the first study that reports reference values for the anterior and posterior regions of the putamen. This may be very useful for early detection of dopaminergic alteration as the posterior putamen was shown to be the most and earliest affected structure of the striatum (Tatsch and Poepperl 2013).

No differences were found between left and right SBRs for any striatal VOI. However, conflicting reports have been published regarding interhemispheric differences in SBR values. For example, in (Matsuda et al. 2018), the authors have reported greater SBRs on the right side, while others have reported greater SBRs on the left side (e.g., (Varrone et al. 2013)). Others, like us, found no interhemispheric differences in any of the measured SBRs (Kaasinen et al. 2015; Lavalaye et al. 2000).

Similarly, no effect of age or gender was found on the interhemispheric asymmetries or on the C/P ratios. We estimated the average of left and right upper reference limits (mean + 2 × SD) of the C/P ratios to be 1.16 (Flash3D) and 1.13 (FBP). These values are lower than the ones reported in (Nicastro et al. 2016; Varrone et al. 2013) and can better differentiate between normal and PD patients who have much higher C/P ratios due to early putaminal degeneration (Nicastro et al. 2016; Shin et al. 2007; Haapaniemi et al. 2001). Depending on the reconstruction method and on the striatal VOI, mean asymmetry values ranged between 6.26 and 8.81%. A new scan acquired and reconstructed using the same protocols as scans in one of the generated databases should raise the possibility of alteration of the presynaptic dopaminergic system when its left or right C/P ratio, or one of its interhemispheric asymmetries, is two SDs above corresponding sample mean. For example, our results suggest that a normal ^123^I-FP-CIT scan is associated with a striatum asymmetry that is lower than 7.84% (Flash3D) or than 6.26% (FBP). In general, normal ^123^I-FP-CIT scans should be associated with low asymmetry values and with C/P ratios that are closer to unity, irrespective of age and gender. Contrary to results reported in (Matsuda et al. 2018), our results revealed a slight but not statistically significant increase of asymmetry values with advancing age, except for the putamen asymmetry for the Flash3D database. It has been proposed that the asymmetry values and C/P ratios may be used for early detection of PD and to differentiate between various forms of parkinsonism (Nicastro et al. 2016; El Fakhri et al. 2006; Sixel-Döring et al. 2011; Contrafatto et al. 2012; Benítez-Rivero et al. 2013), hence the importance of establishing reference limits for these parameters.

For each regional SBR, we calculated the regression line and the ± 95% PI lines. The regression lines (*y*_r_ = slope × age + intercept) determine age-matched references, and the – 95% PI lines (i.e., lower PI lines given by *y*_l_ = *y*_r_ − 2 × SE) act as normality thresholds. The ^123^I-FP-CIT scan of a patient can be labeled as abnormal if its SBR is 2 × SE or more below age-matched reference. For example, the Flash3D striatum SBR of a 65-year-old patient with an abnormal ^123^I-FP-CIT scan is supposed to be below *y* = (− 0.016 × 65 + 4.028) − 0.8 = 2.19 (see Table 5).

Regarding the effect of gender, we found that women had higher uptake than men in all striatal VOIs, in agreement with some previous studies (Varrone et al. 2013; Matsuda et al. 2018; Eusebio et al. 2012; Kaasinen et al. 2015; Lavalaye et al. 2000). However, we found that this difference was only significant for the Flash3D database. The lack of such statistical significance for the FBP database may either be due to the fact that there is no correlation between gender and DAT availability or that such gender difference exists but was not statistically significant due to inaccuracies in the Chang attenuation correction method which uses a simpler approximation to individual head geometry and assumes a uniform attenuation inside the head. This may have led to more statistical noise and then to higher *p* values compared to using CT-based attenuation correction for the Flash3D database. Other factors differentiating the two databases, such as scatter correction, may have led to the discrepancy seen in gender effect. Some published studies have reported higher DAT availability in women compared to men (Lavalaye et al. 2000; Staley et al. 2001; Mozley et al. 2001), whereas others did not report any gender-related difference (Ryding et al. 2004; van Dyck et al. 1995; van Dyck et al. 2002). Various hypotheses were put forward as to why women exhibit higher striatal uptake than men, related either to higher DAT density in women compared to men, or to the differences in the striatal volume between genders (Varrone et al. 2013; Eusebio et al. 2012). In this study, we observed a greater separation between regression lines of men and women SBR data at earlier decades and even more so for the Flash3D database. For both databases, we have established reference values identical for men and women.

In comparison with the ENC-DAT study (Varrone et al. 2013), we found that Flash3D reference values are comparable to those generated by the BRASS method from uncalibrated and ACSC data in the ENC-DAT study for both men and women. For example, the 95% CI mean slope for striatum in men is − 0.015 (range − 0.019 to − 0.011) in the Flash3D database versus − 0.015 (range − 0.021 to − 0.09) in the ENC-DAT study. For women, we found a mean slope of − 0.018 (range − 0.024 to − 0.013) in the Flash3D database versus − 0.018 (range − 0.025 to − 0.011) in the ENC-DAT database. Slight differences between regression parameters in the two databases may be attributed in part to the difference in attenuation correction methods used in both studies. In addition, our mean C/P ratios are uniform across genders and less variable compared to those from the ENC-DAT uncalibrated ACSC data. Note that the comparisons between our study and the ENC-DAT study may be affected by several factors, including methodological differences in acquisition of scans; their reconstruction, correction, and quantification; and most importantly by the differences between the two considered cohorts. However, using data from patients with normal ^123^I-FP-CIT scans who were adequately followed to confirm that they have not developed any form of degenerative parkinsonism, and scanned at a single site following the same protocol, we were able to generate reference values comparable to those generated from healthy volunteers. Equally important is that our mean values for caudate and putamen SBRs in men and women were similar if not identical to those from the ENC-DAT study using BRASS analysis of uncalibrated ACSC data.

Our data also showed that comorbidities (e.g., diabetes, hypertension) did not seem to increase the variance in outcomes when compared to healthy subjects, making it possible for clinicians to build their own normal ^123^I-FP-CIT databases from scans acquired during routine clinical practice. In comparison with a published database generated from patients with normal ^123^I-FP-CIT scans and no degenerative conditions (Nicastro et al. 2017; Nicastro et al. 2016), we have used a larger cohort (237 vs. 182) with a well-balanced gender distribution and a wider age range, including a relatively very young subset of participants. We also had longer follow-ups for all of our subjects who were included through a stringent two-phase selection process. This may have contributed to generating reference values and limits comparable to those of healthy volunteers.

There are some limitations to this study. First, the diagnoses were mainly based on clinical criteria without autopsy confirmation. However, the non-degenerative nature of parkinsonism was assessed by the clinical evolution for 3 to 10 years (4.8 ± 1.3 years), which represents the gold standard to establish a diagnosis in routine clinical practice. Second, our approach for normative data definition was monocentric, which may limit the ability to extend our references to other groups and centers. Effort is underway to generalize the use of the reconstructed databases for other imaging systems and/or other reconstruction/correction methods. This warrants a thorough investigation on how the reconstruction and scanner characterization impact the performance of our reference values in assessing clinical scan normality. An evaluation of the generated reference values against a large sample of degenerative cases is also warranted but is beyond the scope of this paper. Third, we have found that differences in SBRs between genders were only statistically significant for the Flash3D database. We have postulated that the lack of such significance for the FBP database is mainly due to inaccuracies in Chang attenuation correction method which has been shown to be a challenging and often inconsistent task with DAT imaging due to low tracer binding in cortical areas (Barnden et al. 2006). This deserves further investigation. The use of CTAC for the Flash3D database, although it comes at the expense of exposing patients to additional radiation (~ 2 mSv), has the advantage of providing more accurate attenuation correction. This has led to a better separation between men and women SBRs. The importance of this separation may not be an enough justification for the additional radiation exposure. However, the availability of a CT scan had two other major advantages in our approach: (1) the anatomical information provided by the CT scan helped greatly in the diagnostic interpretation of the ^123^I-FP-CT images, in particular for patients with atypical parkinsonian syndromes which are complicated and often involve different pathologies, and (2) the use of a CT scan plays an important role in the semi-quantification pipeline especially for the spatial normalization of ^123^I-FP-CT images that are often very noisy and hard to directly register to a standard template. In this study, CT-based registration was required for 26/237 (11%) cases for which the SPECT-based alignment failed. Finally, the similarity of our results with the results from large multi-center and multi-system studies with healthy volunteers and using similar reconstruction/corrections methods suggests that the ^123^I-FP-CIT binding as a function of age is mostly dependent on the biology of DAT availability. Given the high reproducibility and reliability of the ^123^I-FP-CIT scan (Booij et al. 1998), better understanding of how inter-subject differences affect the relationship between DAT availability, as measured by ^123^I-FP-CIT, and age would require longitudinal studies which are ethically and economically difficult to design and perform.

## Conclusion

This study provides a large database of ^123^I-FP-CIT reference parameters from a cohort of patients with normal SPECT scans and no degenerative parkinsonism, scanned at the same center on the same imaging system and following the same protocol. The generated age-matched SBRs as well as C/P and asymmetry reference values and limits compared favorably to parameters from a normal database of healthy volunteers. Our results could be used to assess new scans acquired and processed in an equivalent manner as one of the reconstructed databases. Generalization to assess scans acquired with other imaging systems and processed with other reconstruction/correction methods is currently under way.

## Data Availability

The datasets generated and/or analyzed during the current study are not publicly available due institutional restrictions on patient confidentiality, prior consent, and privacy.

## Abbreviations

^123^I-FP-CIT: Iodine 123-radiolabeled 2β-carbomethoxy-3β-(4-iodophenyl)-*N*-(3-fluoropropyl) nortropane
AC: Attenuation correction
ACSC: Attenuation and scatter corrections
AD: Alzheimer’s disease
ADHD: Attention deficit hyperactivity disorder
C/P: Caudate-to-putamen ratio
CI: Confidence interval
CTAC: CT-based attenuation correction
DAT: Dopamine active transporter
DIP: Drug-induced parkinsonism
DLB: Dementia with Lewy body
ENC-DAT: European Normal Control Database of DaTscan
ET: Essential tremor
FBP: Filtered back projection
MNI: Montreal Neurological Institute
MSA-C: Cerebellar multiple system atrophy
OSEM-3D: Three-dimensional iterative method based on ordered subset expectation maximization
PD: Idiopathic Parkinson’s disease
PI: Prediction interval
SBR: Specific binding ratio
SD: Standard deviation
SE: Standard error of regression model
SPECT: Single photon emission computed tomography
SWEDD: Scan without evidence of dopaminergic deficit

## Footnotes

1 Wellcome Department of Cognitive Neurology, London, UK

2 In *syngo*.via® software, we report distribution volume ratios (DVRs) instead of SBRs, where DVR *= C*_voi_/*C*_occip_ = SBR + 1. We also report putamen-to-caudate ratio instead of C/P ratio.

## References

[CR1] Albert NL, Unterrainer M, Diemling M, Xiong G, Bartenstein P, Koch W, Varrone A, Dickson JC, Tossici-Bolt L, Sera T, Asenbaum S (2016). Implementation of the European multicentre database of healthy controls for [123 I] FP-CIT SPECT increases diagnostic accuracy in patients with clinically uncertain parkinsonian syndromes. Eur J Nucl Med Mol Imaging..

[CR2] Barnden LR, Dickson J, Hutton BF (2006). Detection and validation of the body edge in low count emission tomography images. Comput Methods Programs Biomed..

[CR3] Benítez-Rivero S, Marín-Oyaga VA, García-Solís D, Huertas-Fernández I, García-Gómez FJ, Jesús S, Cáceres MT, Carrillo F, Ortiz AM, Carballo M, Mir P (2013). Clinical features and 123I-FP-CIT SPECT imaging in vascular parkinsonism and Parkinson’s disease. J Neurol Neurosurg Psychiatry..

[CR4] Booij J, Andringa G, Rijks LJ, Vermeulen RJ, De Bruin K, Boer GJ, Janssen AG, Van Royen EA (1997). [123I] FP-CIT binds to the dopamine transporter as assessed by biodistribution studies in rats and SPECT studies in MPTP-lesioned monkeys. Synapse..

[CR5] Booij J, Habraken JB, Bergmans P, Tissingh G, Winogrodzka A, Wolters EC, Janssen AG, Stoof JC, Van Royen EA (1998). Imaging of dopamine transporters with iodine-123-FP-CIT SPECT in healthy controls and patients with Parkinson’s disease. J Nucl Med.

[CR6] Booij J, Speelman JD, Horstink MW, Wolters EC (2001). The clinical benefit of imaging striatal dopamine transporters with [123I] FP-CIT SPET in differentiating patients with presynaptic Parkinsonism from those with other forms of Parkinsonism. Eur J Nucl Med..

[CR7] Booij J, Tissingh G, Boer GJ, Speelman JD, Stoof JC, Janssen AG, Wolters EC, Van Royen EA (1997). [123I] FP-CIT SPECT shows a pronounced decline of striatal dopamine transporter labelling in early and advanced Parkinson’s disease. J Neurol Neurosurg Psychiatry..

[CR8] Booth TC, Nathan M, Waldman AD, Quigley AM, Schapira AH, Buscombe J (2015). The role of functional dopamine-transporter SPECT imaging in parkinsonian syndromes, part 2. Am J Neuroradiol.

[CR9] Buchert R, Hutton C, Lange C, Hoppe P, Makowski M, Bamousa T, Platsch G, Brenner W, Declerck J (2016). Semiquantitative slab view display for visual evaluation of 123I-FP-CIT SPECT. Nucl Med Commun.

[CR10] Buchert R, Kluge A, Tossici-Bolt L, Dickson J, Bronzel M, Lange C, Asenbaum S, Booij J, Kapucu LÖ, Svarer C, Koulibaly PM (2016). Reduction in camera-specific variability in [123 I] FP-CIT SPECT outcome measures by image reconstruction optimized for multisite settings: impact on age-dependence of the specific binding ratio in the ENC-DAT database of healthy controls. Eur J Nucl Med Mol Imaging..

[CR11] Catafau AM, Tolosa E (2004). Impact of dopamine transporter SPECT using 123I-ioflupane on diagnosis and management of patients with clinically uncertain Parkinsonian syndromes. Mov Disord.

[CR12] Cohen P, West SG, Aiken LS (2014) Applied multiple regression/correlation analysis for the behavioral sciences. Psychology Press, Taylor and Francis Group, New York

[CR13] Contrafatto D, Mostile G, Nicoletti A, Dibilio V, Raciti L, Lanzafame S, Luca A, Distefano A, Zappia M (2012). [123I] FP-CIT-SPECT asymmetry index to differentiate Parkinson’s disease from vascular parkinsonism. Acta Neurol Scand.

[CR14] Costa A, la Fougère C, Pogarell O, Möller HJ, Riedel M, Ettinger U (2013). Impulsivity is related to striatal dopamine transporter availability in healthy males. Psychiatry Res.

[CR15] Darcourt J, Booij J, Tatsch K, Varrone A, Vander Borght T, Kapucu ÖL, Någren K, Nobili F, Walker Z, Van Laere K (2010). EANM procedure guidelines for brain neurotransmission SPECT using 123I-labelled dopamine transporter ligands, version 2. Eur J Nucl Med Mol Imaging..

[CR16] Dickson JC, Tossici-Bolt L, Sera T, De Nijs R, Booij J, Bagnara MC, Seese A, Koulibaly PM, Akdemir UO, Jonsson C, Koole M (2012). Proposal for the standardisation of multi-centre trials in nuclear medicine imaging: prerequisites for a European 123 I-FP-CIT SPECT database. Eur J Nucl Med Mol Imaging..

[CR17] Djang DS, Janssen MJ, Bohnen N, Booij J, Henderson TA, Herholz K, Minoshima S, Rowe CC, Sabri O, Seibyl J, Van Berckel BN (2012). SNM practice guideline for dopamine transporter imaging with 123I-ioflupane SPECT 1.0. J Nucl Med..

[CR18] El Fakhri G, Habert MO, Maksud P, Kas A, Malek Z, Kijewski MF, Lacomblez L (2006). Quantitative simultaneous 99m Tc-ECD/123 I-FP-CIT SPECT in Parkinson’s disease and multiple system atrophy. Eur J Nucl Med Mol Imaging.

[CR19] Eusebio A, Azulay JP, Ceccaldi M, Girard N, Mundler O, Guedj E (2012). Voxel-based analysis of whole-brain effects of age and gender on dopamine transporter SPECT imaging in healthy subjects. Eur J Nucl Med Mol Imaging..

[CR20] Haapaniemi TH, Ahonen A, Torniainen P, Sotaniemi KA, Myllylä VV (2001). [123I] β-CIT SPECT demonstrates decreased brain dopamine and serotonin transporter levels in untreated parkinsonian patients. Mov Disord.

[CR21] Kaasinen V, Joutsa J, Noponen T, Johansson J, Seppänen M (2015). Effects of aging and gender on striatal and extrastriatal [123I] FP-CIT binding in Parkinson’s disease. Neurobiol Aging..

[CR22] Koch W, Radau PE, Münzing W, Tatsch K (2006). Cross-camera comparison of SPECT measurements of a 3-D anthropomorphic basal ganglia phantom. Eur J Nucl Med Mol Imaging..

[CR23] Lavalaye J, Booij J, Reneman L, Habraken JB, van Royen EA (2000). Effect of age and gender on dopamine transporter imaging with [123 I] FP-CIT SPET in healthy volunteers. Eur J Nucl Med..

[CR24] Matsuda H, Murata M, Mukai Y, Sako K, Ono H, Toyama H, Inui Y, Taki Y, Shimomura H, Nagayama H, Tateno A (2018). Japanese multicenter database of healthy controls for [123 I] FP-CIT SPECT. Eur J Nucl Med Mol Imaging..

[CR25] Mozley LH, Gur RC, Mozley PD, Gur RE (2001). Striatal dopamine transporters and cognitive functioning in healthy men and women. Am J Psychiatry..

[CR26] Mozley PD, Kim HJ, Gur RC, Tatsch K, Muenz LR, McElgin WT, Kung MP, Mu M, Myers AM, Kung HF (1996). Iodine-123-IPT SPECT imaging of CNS dopamine transporters: nonlinear effects of normal aging on striatal uptake values. J Nucl Med..

[CR27] Neumeyer JL, Campbell A, Wang S, Gao Y, Milius RA, Kula NS, Baldessarini RJ, Zea-Ponce Y, Baldwin RM, Innis RB (1994). N-Omega-fluoroalkyl analogs of (1R)-2β-carbomethoxy-3β-(4-iodophenyl) tropane (β-CIT): radiotracers for positron emission tomography and single photon emission computed tomography imaging of dopamine transporters. J Med Chem.

[CR28] Nicastro N, Burkhard PR, Garibotto V (2018). Scan without evidence of dopaminergic deficit (SWEDD) in degenerative parkinsonism and dementia with Lewy bodies: a prospective study. J Neurol Sci.

[CR29] Nicastro N, Garibotto V, Allali G, Assal F, Burkhard PR (2017). Added value of combined semi-quantitative and visual [123I] FP-CIT SPECT analyses for the diagnosis of dementia with Lewy bodies. Clin Nucl Med.

[CR30] Nicastro N, Garibotto V, Poncet A, Badoud S, Burkhard PR (2016). Establishing on-site reference values for 123 I-FP-CIT SPECT (DaTscan®) using a cohort of individuals with non-degenerative conditions. Mol Imaging Biol.

[CR31] Nobili F, Naseri M, De Carli F, Asenbaum S, Booij J, Darcourt J, Ell P, Kapucu Ö, Kemp P, Varer C, Morbelli S (2013). Automatic semi-quantification of [123 I] FP-CIT SPECT scans in healthy volunteers using BasGan version 2: results from the ENC-DAT database. Eur J Nucl Med Mol Imaging..

[CR32] O’sullivan SS, Williams DR, Gallagher DA, Massey LA, Silveira-Moriyama L, Lees AJ (2008). Nonmotor symptoms as presenting complaints in Parkinson’s disease: a clinicopathological study. Mov Disord..

[CR33] Pirker W, Asenbaum S, Hauk M, Kandlhofer S (2000). Imaging serotonin and dopamine transporters with 123I-beta-CIT SPECT: binding kinetics and effects of normal aging. J Nucl Med..

[CR34] Postuma RB, Berg D, Stern M, Poewe W, Olanow CW, Oertel W, Obeso J, Marek K, Litvan I, Lang AE, Halliday G (2015). MDS clinical diagnostic criteria for Parkinson’s disease. Mov Disord..

[CR35] Rizzo G, Copetti M, Arcuti S, Martino D, Fontana A, Logroscino G (2016). Accuracy of clinical diagnosis of Parkinson disease: a systematic review and meta-analysis. Neurology..

[CR36] Ryding E, Lindström M, Brådvik B, Grabowski M, Bosson P, Träskman-Bendz L, Rosén I (2004). A new model for separation between brain dopamine and serotonin transporters in 123I-β-CIT SPECT measurements: normal values and sex and age dependence. Eur J Nucl Med Mol Imaging..

[CR37] Shin HY, Kang SY, Yang JH, Kim HS, Lee MS, Sohn YH (2007). Use of the putamen/caudate volume ratio for early differentiation between parkinsonian variant of multiple system atrophy and Parkinson disease. J Clin Neurol.

[CR38] Sixel-Döring F, Liepe K, Mollenhauer B, Trautmann E, Trenkwalder C (2011). The role of 123 I-FP-CIT-SPECT in the differential diagnosis of Parkinson and tremor syndromes: a critical assessment of 125 cases. J Neurol.

[CR39] Söderlund TA, Dickson JC, Prvulovich E, Ben-Haim S, Kemp P, Booij J, Nobili F, Thomsen G, Sabri O, Koulibaly PM, Akdemir OU (2013). Value of semiquantitative analysis for clinical reporting of 123I-2-β-carbomethoxy-3β-(4-iodophenyl)-N-(3-fluoropropyl) nortropane SPECT studies. J Nucl Med..

[CR40] Staley JK, Krishnan-Sarin S, Zoghbi S, Tamagnan G, Fujita M, Seibyl JP, Maciejewski PK, O'Malley S, Innis RB (2001). Sex differences in [123I] β-CIT SPECT measures of dopamine and serotonin transporter availability in healthy smokers and nonsmokers. Synapse..

[CR41] Tatsch K, Poepperl G (2013). Nigrostriatal dopamine terminal imaging with dopamine transporter SPECT: an update. J Nucl Med.

[CR42] Thobois S, Prange S, Scheiber C, Broussolle E (2019). What a neurologist should know about PET and SPECT functional imaging for parkinsonism: a practical perspective. Parkinsonism Relat Disord.

[CR43] Tondeur MC, Hambye AS, Dethy S, Ham HR (2010). Interobserver reproducibility of the interpretation of I-123 FP-CIT single-photon emission computed tomography. Nuclear Med Commun.

[CR44] Tossici-Bolt L, Dickson JC, Sera T, De Nijs R, Bagnara MC, Jonsson C, Scheepers E, Zito F, Seese A, Koulibaly PM, Kapucu OL (2011). Calibration of gamma camera systems for a multicentre European 123 I-FP-CIT SPECT normal database. Eur J Nucl Med Mol Imaging..

[CR45] van Dyck CH, Seibyl JP, Malison RT, Laruelle M, Wallace E, Zoghbi SS, Zea-Ponce Y, Baldwin RM, Charney DS, Hoffer PB, Innis RB (1995). Age-related decline in striatal dopamine transporter binding with iodine-123-β-CITSPECT. J Nucl Med..

[CR46] van Dyck CH, Seibyl JP, Malison RT, Laruelle M, Zoghbi SS, Baldwin RM, Innis RB (2002). Age-related decline in dopamine transporters: analysis of striatal subregions, nonlinear effects, and hemispheric asymmetries. Am J Geriatr Psychiatry..

[CR47] Varrone A, Dickson JC, Tossici-Bolt L, Sera T, Asenbaum S, Booij J, Kapucu OL, Kluge A, Knudsen GM, Koulibaly PM, Nobili F (2013). European multicentre database of healthy controls for [123 I] FP-CIT SPECT (ENC-DAT): age-related effects, gender differences and evaluation of different methods of analysis. Eur J Nucl Med Mol Imaging..

[CR48] Volkow ND, Wang GJ, Newcorn J, Fowler JS, Telang F, Solanto MV, Logan J, Wong C, Ma Y, Swanson JM, Schulz K (2007). Brain dopamine transporter levels in treatment and drug naive adults with ADHD. Neuroimage..

[CR49] Walker Z, Costa DC, Walker RW, Shaw K, Gacinovic S, Stevens T, Livingston G, Ince P, McKeith IG, Katona CL (2002). Differentiation of dementia with Lewy bodies from Alzheimer’s disease using a dopaminergic presynaptic ligand. J Neurol Neurosurg Psychiatry..

